# Using Taiwanese Universal Health Insurance Data to Estimate LTC Needs with Machine Learning

**DOI:** 10.1093/geroni/igab046.3650

**Published:** 2021-12-17

**Authors:** Kuan-Ming Chen, Chen-Wei Hsiang, Shiau-Fang Chao, Ming-Jen Lin, Kuan-Ju Tseng, Yu-Hsuan Chou, Ji-Lung Hsieh, Ya-Mei Chen

**Affiliations:** 1 National Bureau of Economic Research, CAMBRIDGE, Massachusetts, United States; 2 National Taiwan University, National Taiwan University, Taipei, Taiwan (Republic of China); 3 National Taiwan University, Taipei City, Taiwan (Republic of China); 4 National Taiwan University, Taipei, Taipei, Taiwan (Republic of China); 5 Graduate Institute of Journalism, Taipei City, Taipei, Taiwan (Republic of China)

## Abstract

One of the core issues in long-term care (LTC) policy is the growing imbalance between demand and supply of LTC services due to aging population. To estimate the imbalance and allocate LTC resources, the government regularly conducts surveys. These surveys are expensive given the sample size requirements and imprecise given their subjective nature. This study links the administrative records of the universal health insurance database with LTC program usage records in Taiwan to explore this issue. Machine learning algorithms are used in projecting LTC needs from administrative records. LTC program usage records provide detailed LTC needs information and the amount of service each individual used. In addition, health insurance claim data provides rich health information. Specific LTC needs are predicted for each individual. By further extrapolating to future demographics, long-term LTC needs could be projected. There are several findings in this study. Prediction of difficulties in activities of daily livings (ADL), measured by Barthel index, works best using the Gradient Boosting algorithm. The mean absolute error is 17.67 out of a 0 to 100 scale. In addition to dementia and stroke, diagnosis of pressure ulcer (ICD 10 code: L89) and pneumonia (ICD 10 code: J18) have high predictive power for LTC needs. Prediction of Instrumental ADL (IADL) also performs well with a mean absolute error 1.31. The prediction model suggests high LTC needs and excess demand as the demographics changing. Our study provides a reliable way of using rich information to estimate future LTC needs without conducting additional costly surveys.

